# A dynamic probabilistic model of the onset and interaction of cardio-metabolic comorbidities on an ageing adult population

**DOI:** 10.1038/s41598-024-61135-x

**Published:** 2024-05-20

**Authors:** Chiara Roversi, Erica Tavazzi, Martina Vettoretti, Barbara Di Camillo

**Affiliations:** 1 Department of Information Engineering, University of Padua, Via Giovanni Gradenigo, 6/b, 35131 Padua, Italy; 2 Department of Comparative Biomedicine and Food Science, University of Padua, Agripolis, Viale dell’Università, 16, 35020 Legnaro (PD), Italy

**Keywords:** Cardio-metabolic comorbidities, Ageing, Dynamic Bayesian networks, Simulation, Disease progression, Biomedical engineering, Risk factors

## Abstract

Comorbidity is widespread in the ageing population, implying multiple and complex medical needs for individuals and a public health burden. Determining risk factors and predicting comorbidity development can help identify at-risk subjects and design prevention strategies. Using socio-demographic and clinical data from approximately 11,000 subjects monitored over 11 years in the English Longitudinal Study of Ageing, we develop a dynamic Bayesian network (DBN) to model the onset and interaction of three cardio-metabolic comorbidities, namely type 2 diabetes (T2D), hypertension, and heart problems. The DBN allows us to identify risk factors for developing each morbidity, simulate ageing progression over time, and stratify the population based on the risk of outcome occurrence. By applying hierarchical agglomerative clustering to the simulated, dynamic risk of experiencing morbidities, we identified patients with similar risk patterns and the variables contributing to their discrimination. The network reveals a direct joint effect of biomarkers and lifestyle on outcomes over time, such as the impact of fasting glucose, HbA1c, and BMI on T2D development. Mediated cross-relationships between comorbidities also emerge, showcasing the interconnected nature of these health issues. The model presents good calibration and discrimination ability, particularly in predicting the onset of T2D (iAUC-ROC = 0.828, iAUC-PR = 0.294) and survival (iAUC-ROC = 0.827, iAUC-PR = 0.311). Stratification analysis unveils two distinct clusters for all comorbidities, effectively discriminated by variables like HbA1c for T2D and age at baseline for heart problems. The developed DBN constitutes an effective, highly-explainable predictive risk tool for simulating and stratifying the dynamic risk of developing cardio-metabolic comorbidities. Its use could help identify the effects of risk factors and develop health policies that prevent the occurrence of comorbidities.

## Introduction

Comorbidity refers to the co-existence of two or more chronic conditions in the same subject. Affecting mainly people aged 50 or older , with the rapid growth of the ageing population^1^ this condition is increasingly widespread: in England, two-thirds of adults aged over 65 are expected to be living with multiple health conditions by 2035. In particular, the number of subjects living with 4 or more diseases is expected to double with respect to the 2015 estimate^2^. A high prevalence of comorbidities is being detected also in other countries, as documented by several studies ranging on all continents^3–7^.

Subjects with comorbidities have multiple and complex medical needs, leading to a worse quality of life and an enhanced death risk, as well as constituting an economic burden for public health^8^. Consequently, the study of chronic disease progression and comorbidity development/interaction constitutes a topic of current interest for public health research^9,10^. In particular, the identification of relationships between risk factors and health outcomes allows the development of models able to predict the evolution of health outcomes over time. Such models can be used to identify the subjects at risk and, accordingly, to design strategies to prevent or delay the onset of ageing-related diseases. Specifically, the identification of modifiable risk factors may allow the planning of targeted interventions which can preserve or enhance the health status of at-risk subjects. Furthermore, since comorbidities are diseases that develop simultaneously or consequently, with the course of one disease heavily influenced by the course of the others^8^, studies that take into account their interaction mechanisms and co-evolution over time are needed.

In this context, predictive models can be useful for modelling the progression to disease onset as well as the interactions among concomitant diseases, based on a set of input features describing the subject’s health status and life behaviours. In the literature, there is a number of predictive models which aim at forecasting ageing-related diseases’ onsets starting from clinical and lifestyle variables. However, despite the importance that comorbidities-related issues have for the population and health system, the majority of the literature models are focused on a single disease at once rather than considering together multiple diseases and their interactions^11–18^. Some literature works consider multiple diseases in their model. However, these studies typically simplify the multi-outcome problem of the prediction of comorbidity onset into a simple regression problem in which the outcome is the number of chronic diseases per patient. Moreover, most of the literature models consider in input only the variables collected at a single patient visit, thus not taking into account the longitudinal data collected during periodic encounters^19–22^. Other works are instead mainly focused on identifying the subjects’ progression trajectories defined as the successive occurrence of comorbidities^23–26^. Although these models are able to longitudinally analyse the development of comorbidities and their interactions, they include in the model only a few variables (the most used are age, sex, and obesity). This limits the possibility of concurrently investigating the relationships between risk factors and health outcomes.

In this work, we propose a dynamic probabilistic model of the onset and interaction of multiple diseases, which overcomes the limitations of literature approaches. Specifically, we develop a dynamic Bayesian network (DBN) focusing on 3 cardio-metabolic comorbidities with a high worldwide prevalence, namely type 2 diabetes (T2D), hypertension, and heart disease. In the literature, DBNs were successfully applied to model the progression to type 1 diabetes complications^27^, diabetes onset^15^, and amyotrophic lateral sclerosis stages^28^. In this work, DBN is applied to simultaneously consider different outcomes (here, the cardio-metabolic comorbidities) and study how the interactions, represented as conditional probabilities, between them and with other potential risk factors change over time. Noticeably, this technique allows for the representation and investigation of the emerging relationships in a highly interpretable way, facilitating the analysis and the communication of results. The proposed model is developed using data from the English Longitudinal Study of Ageing (ELSA), a study that collects multidisciplinary data from a representative sample of the English population mostly aged 50 and older. Specifically, we selected a number of socio-demographic, clinical, health indicators, and blood test variables as potential risk factors for cardio-metabolic comorbidities development. The DBN highlights both the direct and indirect effects of the considered variables on the outcomes, allowing a high explainability of the relationships among all the variables involved in the model. Based on factors with a direct impact on the outcomes, we then perform a stratification analysis to identify the characteristics of the population at major risk of developing each morbidity, thus effectively employing the trained DBN as a simulation tool to perform risk stratification.

## Material and methods

### Material

#### The English longitudinal study of ageing dataset

In this work, we employed the dataset of the ELSA, an ongoing study funded by the U.S. National Institute on Aging and a consortium of UK government departments^29,30^. Collecting data from people aged over 50 and their partners, the ELSA aims at understanding different aspects of ageing in England. The available data include information on subjects’ physical and mental health, well-being, social and economic status, as well as their attitudes around ageing and how these change over time.

Since its beginning in 2002, more than 18,500 people have taken part in the study, with the same subjects re-interviewed about every 2 years, for a total of 10 waves of data collection. To avoid the youngest ages being unrepresented as the study progresses due to the ageing of the participants, the ELSA sample has been refreshed five times, specifically at waves 3, 4, 6, 7 and 9. At each wave, participants were administered interviews conducted face-to-face using computer-assisted interviewing (CAPI) combined with self-completion questionnaires completed using pen and paper (PAPI). Moreover, a CAPI interview and collection of bio-measures by a qualified nurse have been carried out for all participants at waves 2, 4, and 6 and for half of the participants in waves 8 and 9, providing further details on the subjects’ health status.

The design choices and the performed preprocessing are detailed in the following section.

#### Data pre-processing

From the ELSA, we considered the harmonised data collected in waves 1–7, which include information on more than 18,500 subjects. According to the collection design, we observed that only the even waves include bio-measures such as the results of blood tests (called biomarkers from here on) collected in clinical examinations. Therefore, in order to reduce the systematic missingness of the data at the first observation time point, we removed wave 1 from our analysis. Then, in order to fix the temporal step between the interview appointments equal to the step between the waves (about 2 years) for all the participants, we selected for each subject only their consecutive visits within waves 2–7. Among these visits, the first participated one for each subject was labelled as the *baseline*. This resulted in subjects observed for a maximum of 6 consecutive visits, up to 11 years from baseline.

Then, from the entire pool of participants, we selected the subjects: (i) with at least two available visits, to be able to depict their ageing dynamics; (ii) with the age at baseline greater than 40 years, to focus the analysis on the ageing process in the older, most represented group; and (iii) without all biomarkers missing, to avoid including subjects with systematic missingness of blood test variables. A total of 11,160 subjects satisfied these conditions and were thus included in the final study dataset.

We considered as outcomes for our model the three cardio-metabolic morbidities—namely, T2D, hypertension, and cardiovascular problems—that are included in the data as binary variables (absence/presence of each morbidity) collected at each wave. Specifically, a subject is considered to have developed T2D if a positive answer is given to the question “Has a doctor ever told you that you have diabetes or high blood sugar?” at any follow-up visit. Similar questions are raised for the other two diseases considered in our analysis, namely consisting in “Has a doctor ever told you that you have high blood pressure or hypertension?” and “Has a doctor ever told you that you have heart problems such as angina, heart attack, congestive heart failure, a heart murmur, an abnormal heart rhythm, or others?”.

We also included in the analysis the survival information of the subjects, coded as a binary variable computed looking at the year of death reported in the ELSA, indicating if the subject dies within 4 years from the visit.

Among the wide number of variables constituting the ELSA dataset, we then selected the ones potentially related to the outcomes of interest. Table 1 details these variables that include socio-demographic characteristics, lifestyle habits, two health indicators that represent difficulty in Activity of Daily Living (ADL) and difficulty in Instrumental Activity of Daily Living (IADL), together with some physical measurements and biomarkers. These variables include some static information related to subjects’ stationary characteristics or demographics, such as the subjects’ sex, age at the baseline wave, and level of education; the other considered variables are dynamic, which means that they were collected in all the participated waves to monitor possible changes over time. Moreover, categorical, ordinal, and continuous variables are present: for instance, the history of smoking habits can assume 3 different values depending if the subject has ever smoked in the past, is a current smoker, or never smoked; on the other hand, the ADL and IADL scores assume discrete values on a scale ranging from 0 to 5, where a lower value indicates an increased need of support; conversely, biomarkers can only assume continuous values. Finally, to detect the time passed from baseline, we introduced for each visit an additional time variable, namely Time Since Baseline (TSB).

**Table 1:** Variables included in the model with the corresponding category, name, description, and values or units of measurement for categorical and continuous variables, respectively.

Category	Variable name	Description	Values/Unit of measurement
Socio- demographics	Sex	Subject sex	Male/Female
	Baseline age	Age at baseline interview	[years]
	Education	Education level	Less than high-school/High-school graduate/College or more
	Marital status	Civil status	Married or civil partner/Separated, divorced or widowed/Never married
	Retirement	Has the subject retired?	Yes/No
Lifestyle	Physical activity	Frequency of moderate or vigorous physical activity	Hardly ever or never/1-3 per month or 1 per week/More than 1 per week
	Smoking	Has the subject ever smoked?	Never/Past/Current
	Drinking	Has the subject had an alcoholic drink during the last 12 months?	Yes/No
	Drink count	Number of days the subject had an alcoholic drink in the last week	[days]
Health indicators	ADL	Difficulty in Activity of Daily Living: bathing, dressing, eating, walking across room, getting in/out of bed	[0–5 score]
	IADL	Difficulty in Instrumental Activity of Daily Living: using phone, managing money, taking medications, shopping for groceries, preparing hot meals	[0–5 score]
Physical measurements	BMI	Body mass index	[kg/m^2^]
	Systolic BP	Systolic blood pressure	[mmHg]
	Diastolic BP	Diastolic blood pressure	[mmHg]
Biomarkers	Fasting glucose	Fasting glucose	[mmol/l]
	HbA1c	Glycated hemoglobin	[%]
	Cholesterol	Total cholesterol	[mmol/l]
	HDL	High-density lipoprotein	[mmol/l]
	LDL	Low-density lipoprotein	[mmol/l]
	Triglycerides	Triglyceride level	[mmol/l]
	Fibrinogen	Fibrinogen level	[g/l]
	CRP	C-Reactive Protein level	[mg/l]
	Ferritin	Ferritin level	[ng/ml]
Time	TSB	Current time since baseline (TSB) interview	[years]
Outcomes	Diabetes	Has the subject experienced Type 2 Diabetes onset until now?	Yes/No
	Hypertension	Has the subject experienced hypertension onset until now?	Yes/No
	Heart problems	Has the subject experienced heart problems onset until now?	Yes/No
	Survival	Will death occur within 4 years from this interview?	Yes/No

Of the entire set of subjects, 1476 of them reported having diabetes in at least one of the follow-up visits. Similarly, 5358 subjects reported to have hypertension, while 2836 declared cardiovascular problems.

We then split the data into a training set (75% of the subjects, corresponding to 8328 subjects and 37,085 total visits) and a test set (25% of the subjects, corresponding to 2832 subjects and 12,620 visits), by stratifying for the subjects’ age at baseline. This resulted in a well-stratified split also with respect to the other variables included in the model. Table 2 reports an overview of the full dataset and of the training and test set, based on the characterisation of the subjects at their baseline wave, together with the p-values of the Kruskal-Wallis and $\chi ^2$ tests that we performed on the continuous and categorical variables, respectively, to verify the stratification of the variables in the imputed training and test sets. Tables 3 and 4 report the cardinality of each outcome among the subjects for each of their included waves, starting from the baseline one, in the training and test sets, respectively.

**Table 2:** Demographic and clinical features at baseline of the ELSA population included in the study.

Variable		Full dataset (n=11160)	Training set (n=8328)	Test set (n=2832)	p-value
Sex	Male	5028 (45.1%)	3730 (44.8%)	1298 (45.8%)	0.26
	Female	6132 (54.9%)	4598 (55.2%)	1534 (54.2%)	
Baseline age		63.15±9.78	63.18±9.81	63.07±9.68	0.67
Education	Less than high-school	5995 (53.7%)	4467 (53.6%)	1528 (54.0%)	0.15
	High-school graduate	1761 (15.8%)	1341 (16.1%)	420 (14.8%)	
	College or more	3404 (30.5%)	2520 (30.3%)	884 (31.2%)	
Marital Status	Married or civil partner	7566 (67.8%)	5657 (67.9%)	1909 (67.4%)	0.58
	Separated, divorced or widowed	2922 (26.2%)	2164 (26.0%)	758 (26.8%)	
	Never married	672 (6.0%)	507 (6.1%)	165 (5.8%)	
Retirement	Yes	4887 (43.8%)	3641 (43.7%)	1246 (44.0%)	0.77
	No	6273 (56.2%)	4687 (56.3%)	1586 (56.0%)	
Physical activity	Hardly ever or never	1545 (13.8%)	1149 (13.8%)	396 (14.0%)	0.61
	1-3 per month or 1 per week	2241 (20.1%)	1688 (20.3%)	553 (19.5%)	
	More than 1 per week	7374 (66.1%)	5491 (65.9%)	1883 (66.5%)	
Smoking	Never	4383 (39.3%)	3251 (39.0%)	1132 (40.0%)	0.33
	Past	5034 (45.1%)	3786 (45.5%)	1248 (44.1%)	
	Current	1743 (15.6%)	1291 (15.5%)	452 (16.0%)	
Drinking	Yes	9983 (89.5%)	7467 (89.7%)	2516 (88.8%)	0.15
	No	1177 (10.5%)	861 (10.3%)	316 (11.2%)	
Drink count		2.36±2.43	2.35±2.43	2.39±2.44	0.51
ADL		0.30±0.80	0.30±0.80	0.30±0.78	0.75
IADL		0.16±0.55	0.16±0.55	0.16±0.55	0.89
BMI		28.13±5.08	28.14±5.07	28.12±5.10	0.95
Systolic BP		133.22±17.95	133.16±17.96	133.39±17.91	0.63
Diastolic BP		75.27±10.78	75.25±10.82	75.30±10.67	0.88
Fasting glucose		4.99±0.77	4.99±0.77	5.00±0.76	0.19
HbA1c		5.42±1.21	5.41±1.23	5.43±1.17	0.32
Cholesterol		5.79±1.10	5.80±1.10	5.79±1.10	0.44
HDL		1.54±0.38	1.54±0.38	1.54±0.38	0.70
LDL		3.45±0.94	3.45±0.94	3.45±0.94	0.88
Triglycerides		1.72±1.07	1.72±1.07	1.71±1.06	0.27
Fibrinogen		3.19±0.61	3.19±0.61	3.20±0.63	0.49
CRP		3.47±6.99	3.50±7.05	3.39±6.82	0.67
Ferritin		121.38±112.42	122.32±117.55	118.64±95.74	0.32
Diabetes	Yes	794 (7.1%)	599 (7.2%)	195 (6.9%)	0.53
	No	10366 (92.9%)	7729 (92.8%)	2637 (93.1%)	
Hypertension	Yes	3982 (35.7%)	2944 (35.4%)	1038 (36.7%)	0.15
	No	7178 (64.3%)	5384 (64.6%)	1794 (63.3%)	
Heart problems	Yes	1614 (14.5%)	1216 (14.6%)	398 (14.1%)	0.41
	No	9546 (85.5%)	7112 (85.4%)	2434 (85.9%)	
Survival	Yes	391 (3.5%)	294 (3.5%)	97 (3.4%)	0.82
	No	8884 (79.6%)	6641 (79.7%)	2243 (79.2%)	
	NA	1885 (16.9%)	1393 (16.7%)	492 (17.4%)	

Kruskal-Wallis and $\chi ^2$ tests at 0.05 significance level were used for assessing the equality of the distributions of the continuous and the categorical variables, respectively, in the training and independent test sets.

**Table 3:** Cardinality (percentage) of each outcome among the subjects for each of their included wave, starting from the baseline one, in the training set.

Outcome		1st included wave (baseline) (n=8328)	2nd included wave (n=8328)	3rd included wave (n=6962)	4th included wave (n=6109)	5th included wave (n=4212)	6th included wave (n=3146)
Diabetes	Yes	599 (7.2%)	741 (8.9%)	732 (10.5%)	751 (12.3%)	531 (12.6%)	428 (13.6%)
	No	7729 (92.8%)	7587 (91.1%)	6230 (89.5%)	5358 (87.7%)	3681 (87.4%)	2718 (86.4%)
Hypertension	Yes	2944 (35.4%)	3349 (40.2%)	3027 (43.5%)	2786 (45.6%)	2030 (48.2%)	1628 (51.7%)
	No	5384 (64.6%)	4979 (59.8%)	3935 (56.5%)	3323 (54.4%)	2182 (51.8%)	1518 (48.3%)
Heart problems	Yes	1216 (14.6%)	1445 (17.4%)	1334 (19.2%)	1339 (21.9%)	989 (23.5%)	859 (27.3%)
	No	7112 (85.4%)	6883 (82.6%)	5628 (80.8%)	4770 (78.1%)	3223 (76.5%)	2287 (72.7%)
Survival	Yes	294 (3.5%)	525 (6.3%)	388 (5.6%)	154 (2.5%)	0 (0%)	0 (0%)
	No	6641 (79.7%)	4636 (55.7%)	3272 (47%)	220 (3.6%)	0 (0%)	0 (0%)
	NA	1393 (16.7%)	3167 (38%)	3302 (47.4%)	5735 (93.9%)	4212 (100%)	3146 (100%)

**Table 4:** Cardinality (percentage) of each outcome among the subjects for each of their included wave, starting from the baseline one, in the test set.

Outcome		1st included wave (baseline) (n=2832)	2nd included wave (n=2832)	3rd included wave (n=2342)	4th included wave (n=2058)	5th included wave (n=1472)	6th included wave (n=1084)
Diabetes	Yes	195 (6.9%)	257 (9.1%)	252 (10.8%)	249 (12.1%)	183 (12.4%)	156 (14.4%)
	No	2637 (93.1%)	2575 (90.9%)	2090 (89.2%)	1809 (87.9%)	1289 (87.6%)	928 (85.6%)
Hypertension	Yes	1038 (36.7%)	1197 (42.3%)	1072 (45.8%)	986 (47.9%)	741 (50.3%)	585 (54%)
	No	1794 (63.3%)	1635 (57.7%)	1270 (54.2%)	1072 (52.1%)	731 (49.7%)	499 (46%)
Heart problems	Yes	398 (14.1%)	471 (16.6%)	432 (18.4%)	422 (20.5%)	329 (22.4%)	286 (26.4%)
	No	2434 (85.9%)	2361 (83.4%)	1910 (81.6%)	1636 (79.5%)	1143 (77.6%)	798 (73.6%)
Survival	Yes	97 (3.4%)	177 (6.3%)	114 (4.9%)	38 (1.8%)	0 (0%)	0 (0%)
	No	2243 (79.2%)	1605 (56.7%)	1132 (48.3%)	67 (3.3%)	0 (0%)	0 (0%)
	NA	492 (17.4%)	1050 (37.1%)	1096 (46.8%)	1953 (94.9%)	1472 (100%)	1084 (100%)

We observed the presence of missing data for several variables, ranging from a few observations for some socio-demographic and lifestyle variables such as the level of physical activity and smoking, to around 60% of the total observations for the biomarkers. It is important to underline that the high missing rate detected for biomarkers is due to the specific design of the study, for which such variables were collected only every 4 years and not every 2 years as the others. To not fully discard this precious information, before implementing the network we imputed the missing values to obtain a complete dataset. We designed an algorithm that combines different imputation strategies to deal with the different types of variables and different situations that were present in the data. For the dynamic variables, that are collected for each subject over their consecutive visits, we imputed the missing data employing the information available in the subject’s other waves. Specifically, missing values that were between two neighbouring available data were filled by applying a linear interpolation approach, while the missing data that were present at the beginning or at the tail of the time series were replaced by employing a Next Observation Carried Backward (NOCB) or a Last Observation Carried Forward (LOCF) strategy, respectively. When interpolation could not be performed, i.e. when just one single value was available for a continuous dynamic variable or to replace a missing categorical dynamic feature, the propagation of the next or last value (i.e., NOCB or LOCF) was directly employed. Finally, for those subjects without any recording of a specific dynamic variable, as well as to impute the static features of the dataset, the missing values were replaced with the population median or mode (for the continuous and categorical features, respectively) of that variable computed on the training data. This imputation algorithm was applied to all the variables considered in the network, except for: baseline age, sex, and TSB, which had no missing values; and the variables considered as final outcomes, i.e., the ones related to diabetes, hypertension, cardiovascular problems, and death occurrence.

Considering the mixed type of the selected variables, we decided to employ a discrete-time, discrete-space implementation of the DBN, which represents probabilistic associations between discrete variables over a finite number of time steps (here, the waves). Accordingly, we discretised the continuous variables by employing the quantisation thresholds reported in Table 5. Specifically, common clinical thresholds were used to quantise the variables related to blood tests and physical measurements, while the tertiles of the distribution across the training patients were employed for baseline age. Moreover, manually selected thresholds were used for unbalanced variables such as drink count, ADL, and IADL, in order to reduce unbalanced categories. Finally, given that the maximum observed follow-up on the population is 11 years (our preprocessed data comprise 6 waves, from 2 to 7, at a step of approximately 2 years each), a quantisation interval of 4 years was chosen for the TSB (i.e. quantisation thresholds at 4 and 8 years) to cut the distribution of this variable into three parts with approximately equal time durations. In this way, a similar number of discretisation levels were considered for all variables included in the model.

**Table 5:** Quantisation levels of the continuous variables included in the study and corresponding values.

Variable	Quantisation levels	Quantised values	Criterion
Baseline age	< 55	0	Tertiles
	[55, 65[	1	
	$\ge$ 65	2	
Drink count	< 3	0	Manual
	$\ge$ 3	1	
ADL	< 1	0	Manual
	$\ge$ 1	1	
IADL	< 1	0	Manual
	$\ge$ 1	1	
BMI	< 25	0	^31^
	[25, 30[	1	
	$\ge$ 30	2	
Systolic BP	< 120	0	^32^
	[120, 140[	1	
	$\ge$ 140	2	
Diastolic BP	< 60	0	^32^
	[60, 90[	1	
	$\ge$ 90	2	
Fasting glucose	< 5.6	0	^33^
	$\ge$ 5.6	1	
HbA1c	< 6.5	0	^34^
	$\ge$ 6.5	1	
Cholesterol	< 5.2	0	^35^
	$\ge$ 5.2	1	
HDL	< 1.3	0	^35^
	$\ge$ 1.3	1	
LDL	< 3.4	0	^35^
	$\ge$ 3.4	1	
Triglycerides	< 1.7	0	^36^
	$\ge$ 1.7	1	
Fibrinogen	< 4	0	^37^
	$\ge$ 4	1	
CRP	< 1	0	^38^
	[1, 3[	1	
	$\ge$ 3	2	
Ferritin	< 200	0	^39^
	$\ge$ 200	1	
TSB	< 4	0	Manual
	[4, 8[	1	
	$\ge$ 8	2	

For each variable, the used quantisation criterion is reported; for the variables that have been quantised based on clinical thresholds, a reference is indicated.

### Methods

#### Dynamic Bayesian networks

As a modelling technique, we employed DBNs. A DBN is a probabilistic graphical model of dynamic stochastic processes, where a set of random variables and their dependencies over adjacent time steps are represented as a Directed Acyclic Graph (DAG)^40^. A DAG consists of nodes and directed edges, which correspond to random variables and the influence – namely a conditional probability – of “parent” nodes on their “child” nodes, respectively. Relationships among variables are described by conditional probability tables (CPTs) of each variable at time *t* given the values of its parents at the previous time step $t-1$. In this work, we considered discrete-time DBNs, meaning that each variable has a finite, discrete number of states and the time index *t* increases by one when considering a new observation, here represented by a subject’s new wave.

The learning of the DBN was performed on the training set by employing the R package *bnstruct*^41^, which permits learning the network’s structure and parameters on discrete data. The DBN structure was learned using the Maximum Minimum Hill-Climbing (MMHC) algorithm, which first employs conditional independence testing to identify the skeleton of the network (i.e., the parents and children set of variables), then orients it using a greedy hill-climbing search in the space of the networks^42^. The Bayesian Information Criterion (BIC)^43^ was used in this work to score the different network structures during the search. Once the DBN structure is learned, the maximum *a posteriori* estimation is used to compute the CPTs.

It has to be noticed that, in general, learning an optimal DBN structure is an NP-hard task. To limit the search space, some constraints can be imposed on the network in the learning phase by, for instance, providing some a priori knowledge. In practice, such knowledge is given to the learning algorithm by coding a layering of the variables, i.e., by organising variables in different groups with different rules among them. This arrangement allows excluding any non-sense relationship, such as the unfeasible, retrospective influence of a variable at time *t* on a variable at the previous time step $t-1$, or any non-interesting dependencies, such as the conditional dependency between the subjects’ age and their sex (which, in our case, would basically only describe the relationship between these two characteristics in the study population). In this work, we grouped the variables into 6 layers as reported in Table 6. In general, a variable can have only parents in the upper (i.e. lower-numbered) layers, apart from some exceptions. In our case, the demographic variables in layer 1 (sex and baseline age) were allowed to influence education (layer 2), variables at time *t* (layer 4), and survival (layer 5). Education was allowed to influence variables at time *t* and survival. Variables at time $t-1$ (layer 3) and TSB (layer 6) were not allowed to have parents, but could influence variables at time *t* and survival.

**Table 6:** Layering of the variables imposed on the DBN in the learning phase.

Nr.	Layer name	Variables
1	Demographic	Sex, Baseline age
2	Schooling	Education level
3	Variables at time *t-1*	Marital Status, Retirement, Physical activity, Smoking, Drinking, Drink count, ADL, IADL, BMI, Systolic BP, Diastolic BP, Fasting glucose, HbA1c, Cholesterol, HDL, LDL, Triglycerides, Fibrinogen,CRP, Ferritin, Diabetes, Hypertension, Heart problems
4	Variables at time *t*	Marital Status, Retirement, Physical activity, Smoking, Drinking, Drink count, ADL, IADL, BMI, Systolic BP, Diastolic BP, Fasting glucose, HbA1c, Cholesterol, HDL, LDL, Triglycerides, Fibrinogen, CRP, Ferritin, Diabetes, Hypertension, Heart problems
5	Survival	Survival
6	Time	TSB

As a starting point for the structure search, we provided a network obtained through a 50-fold cross-validation (CV) process on the training set. Specifically, at each iteration, a DBN was learned on a new subset of the training set obtained by merging the data of 49/50 folds, starting from a null network. The 50 different DAGs resulting from this procedure were combined together in a Weighted Partially Directed Acyclic Graph (WPDAG), whose adjacency matrix at position (*i*, *j*) takes the number of occurrences of the edge going from node *i* to node *j* in the 50 so-obtained DAGs. A number of folds equal to 50 was chosen to have both (1) a sufficiently high number of iterations in the CV procedure to assess the presence or absence of an edge (whose estimate improves as the number of folds increases), and (2) a sufficiently high number of observations to learn each DBN (which increases as the number of folds decreases). Such a number of folds also allow us to have a reasonable computational time. Finally, a network with only the most reliable edges, that is, those included in at least 80% of the CV-trained DAGs, was obtained. After checking that the acyclicity assumption was fulfilled, the resulting DAG was employed as the initial network in the final learning process performed on the entire training set.

#### Simulation

From the procedure described above, a network is obtained, which describes the dynamic and static relationships among variables in terms of CPTs and can be used to simulate data starting from an initial condition. We, therefore, designed an algorithm that, for each subject, starting from a complete vector consisting of the values of the variables at his/her baseline visit, uses the learned DBN to simulate the following waves. In the first step, the algorithm takes the subject’s true baseline values and uses the learned network’s structure and CPTs to predict the value of each dynamic child variable at the next time point (set equal to 2 years from the current wave, that is the average time between two training waves) based on the values of its parents at the baseline wave. This procedure is then iterated until a stopping condition is reached, that is, a given time horizon of interest is reached or the in-silico subject dies. In this way, we obtain a sequence of simulated waves, starting from the baseline condition, that constitutes a predicted follow-up.

Since the simulation algorithm is probabilistic, the simulation is run multiple times for each subject to obtain a probabilistic estimate of the predicted follow-up.

#### Model validation

The performance of the learned DBN was assessed by simulating the evolution of test set subjects (employing the simulation algorithm introduced in the previous section) and then comparing the simulated outcomes with the observed ones, in terms of their frequency or time of occurrence. It is important to note that, while the DBN model was learned on the training set, the validation was performed on the test set, thus using data from the baseline visit of subjects never used for training the DBN model (i.e., neither the first visit nor the following visits from these subjects were ever used to learn the model). Since the simulation algorithm requires the input vector, i.e., the baseline visit, to be a complete set of variables, we first reduced the test set to the only subjects with no missing variables at baseline (please notice that, after the imputation, missing values may have remained in the outcome variables only). Next, we ran the simulation 100 times for each subject of the test set. For each simulation, we obtained a predicted follow-up describing how the condition of the subject evolved over time starting from his/her observed initial condition. Then, we extracted the occurrence time of each outcome for both the observed data and the simulated data, by considering as simulated population all the subjects’ repetitions together. We finally compared the distributions of these occurrence times in the observed and simulated follow-up data using the Kaplan-Meier estimator. For a given event (or end-point), the Kaplan-Meier estimator is a non-parametric estimator of the so-called survival curve, describing the probability of being event-free until a certain time for different times. In particular, this estimator takes into account the contribution of the censored subjects, i.e., the subjects whose observed and/or simulated follow-up ended without having observed the end-point. We compared the similarity of the Kaplan-Meier survival curves both by visual inspection and from the statistical point of view using the log-rank test: the more the observed and the simulated survival curves are close to each other, the better the model calibration. In particular, when the p-values of the log-rank test are above the significance threshold (here set equal to 0.05), the two curves are not statistically significantly different. In this case, we can assume that the model is well calibrated, that is, the observed and predicted risks are consistent, with the model neither underestimating nor overestimating the risk.

We further evaluated the DBN performance by assessing its discrimination ability, that is, the capability of the model to predict a longer time to event (or a lower risk) for those subjects who actually experience the endpoint later in reality or who do not experience the event at all in their real follow-up^44^. As discrimination measures, we used the integrated area under (AU) the Receiver Operating Characteristic (iAU-ROC) curve and the integrated Area Under the Precision-Recall curve (iAUC-PR). The higher the values of iAU-ROC and iAUC-PR, which are both in the range 0–1, the better the model’s ability to discriminate. In these computations, we employed as the predicted risk of occurrence of each outcome for each subject the opposite of the area under the Kaplan-Meier curve computed over their 100 repetitions. In detail, we first computed for each clinical outcome the time-dependent AU-ROC and the time-dependent AUC-PR, which allow us to assess the performance of the model over time^45^. The time-dependent AU-ROC and AU-PRC were computed at a 2-year step from the baseline, which represents the average interval among two consecutive real waves, as well as the interval set for the simulation. We computed the AU-ROC and the AUC-PR values up to 8 years for the cardio-metabolic comorbidities, while for death we limited the assessment to 4 years due to the definition given to death (*Will death occur within 4 years from this interview?*) and the real follow-up duration. These thresholds were chosen by observing that the percentage of subjects in the real dataset who experienced the outcomes after the chosen threshold was less than 2.5%. For both of these computations, we used the *PRROC* package in R^46^. Then, for each clinical outcome, we calculated the iAU-ROC and iAUC-PR by averaging over all these time points the time-dependent AU-ROC and AUC-PR, respectively, according to^45^, to obtain an overall model performance.

#### Stratification analysis

A DBN can also be employed to investigate the effect of one or more given variables on an outcome. Specifically, the simulation can be used to forecast subject follow-up of populations/cohorts with diverse characteristics at baseline. Then, the predicted progression can be analysed to study the impact of the different starting values of the clinical features on the risk of outcome occurrence. In this setting, the greatest observable impact is the one on a child variable given different values of one or more of its parents. We thus employed both the subjects of the training and test set together – in order to enhance the observable effect – to investigate the impact of the parent variables on each cardio-metabolic morbidity.

For each morbidity, we stratified the subjects according to the combination of the morbidity’s parents’ values at the first subject’s visit, and simulated each cohort by running 100 repetitions for each subject. Then, we compared the risk of experiencing the morbidity over time among the populations, represented in terms of Kaplan-Meier estimated curves.

To quantitatively assess differences and similarities among the cohorts, we grouped the so-obtained survival curves using hierarchical agglomerative clustering. First, we normalised the curves at each time point between 0 and 1 and computed the Euclidean distance between them. Then, we employed hierarchical clustering with Ward’s clustering criterion as the agglomeration method. Finally, we employed the R package NbClust^47^ to determine the most appropriate number of clusters, considering a range from 2 to 6 possible groups. The method implemented in NbClust is based on the use of 26 validity indices for determining the best number of clusters, each combining information about intra-cluster compactness and inter-cluster isolation, as well as other factors, such as geometric or statistical properties of the data, the number of data objects and dissimilarity or similarity measurements. The optimal number of clusters is then chosen according to the majority rule.

### Ethics approval and consent to participate

All the analyses performed in this work were carried out in accordance with relevant guidelines and regulations. All participants gave written informed consent at the recruitment wave to participate in the ELSA and at each subsequent wave. Ethical approval for all the ELSA waves was granted by NHS Research Ethics Committees under the National Research and Ethics Service (NRES). Information on the ethical approval received for each wave of ELSA can be found at: https://www.elsa-project.ac.uk/ethical-approval, https://www.elsa-project.ac.uk/ethical-approval. The authors assert that all procedures contributing to this work comply with the relevant national and institutional committees’ ethical standards on human experimentation and with the Helsinki Declaration of 1975, as revised in 2008.

## Results

### Network learning

The CV procedure performed on the training set explained in Sect. "Dynamic Bayesian networks" produced a WPDAG with 123 edges, 93 of which were maintained in the DAG obtained by filtering out the less reliable ones (see Sect. S1.1 of the Supplementary Material, Figure S1).

**Figure 1:**
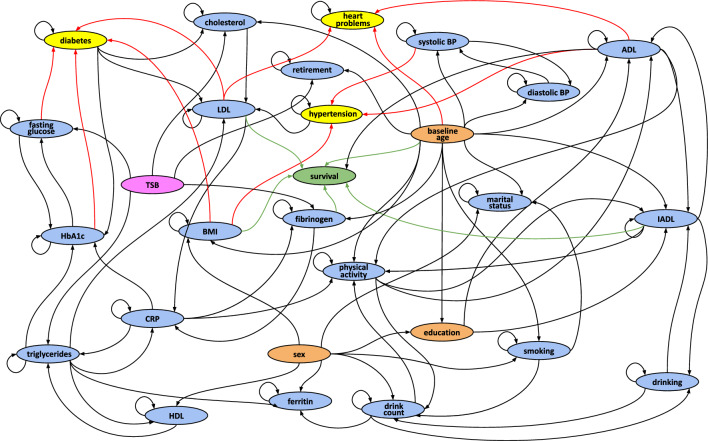
DBN obtained on the training dataset. Static variables are reported in orange, dynamic variables in blue, the time variable in magenta, and the survival in green, while the outcomes, i.e., T2D, hypertension, and heart problems onset, are in yellow. Edges representing a direct influence of a variable on the outcome nodes are marked in red for cardio-metabolic comorbidities, and in green for survival. The loops on the dynamic variables indicate the influence of the variable at a time (t-1) on itself at the subsequent time (t).

By employing this DAG as the starting network for the learning process of the final model, we obtained the final DBN reported in Fig. 1, which includes a total of 101 edges. Graphically, each dynamic variable is represented by a unique node, with loops indicating the influence of the variable at time $t-1$ on itself at subsequent time *t*. Direct edges entering a node represent a joint effect of the parent nodes (at the previous time point, in the case of dynamic variables) on the child node, quantitatively described by a CPT. Specifically, the network highlights a joint direct effect of fasting glucose, HbA1c, BMI, and LDL cholesterol on diabetes onset. BMI also contributes to the development of hypertension, combined with difficulty in ADL and systolic BP, as well as to death, together with fibrinogen levels, difficulty in IADL, baseline age, and LDL cholesterol. Finally, a joint direct effect of LDL cholesterol, baseline age, and difficulty in ADL emerges for what concerns the development of heart problems. The graph structure highlights not only direct dependencies between risk factors and outcomes as the ones mentioned above, but also indirect effects can be observed. For example, the level of moderate or vigorous physical activity has an indirect effect on heart problems, mediated by difficulty in ADL, and on death occurrence, through difficulty in IADL. Furthermore, baseline age and diastolic BP exert an influence on the onset of hypertension, mediated by the level of systolic BP. Interestingly, we can also observe some cross-dependencies among couples of variables such as the above-mentioned relationship between the level of moderate or vigorous physical activity and ADL, which also occurs in the other direction.

### Network performance on the test set

**Figure 2:**
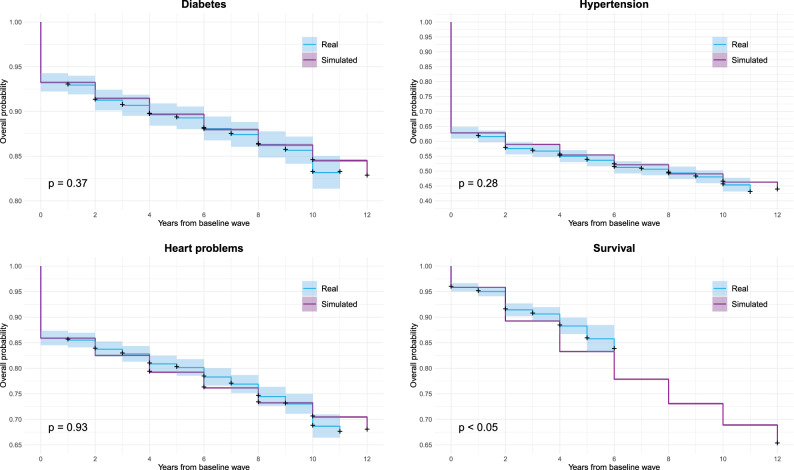
Kaplan-Meier curves of cardio-metabolic comorbidities onset and survival for the test data. Shaded areas denote confidence intervals ($\alpha$ = 0.05), + indicates censored subjects. For each outcome, the log-rank test’s p-value is reported, with a significance threshold set equal to 0.05.

Figure 2 reports the time of occurrence of the real and simulated outcomes computed in terms of the Kaplan-Meier estimated curves in the test set population with a complete baseline visit (n=2340). As can be seen from the figure, in the real population the outcomes have some occurrences at odd years (being the distance between two consecutive real waves only approximately equal to 2 years). However, since the simulation was performed with a time step of 2 years, the visual comparison among the real and simulated Kaplan-Meier curves has to be limited to the common, even time points. We can also observe that the confidence interval of the simulated curve is noticeably narrow, given the increased dimension of the simulated population that includes all the 100 repetitions per patient. Together with the visual inspection, the log-rank test quantitatively confirms that the model is in general well calibrated (all p-values $>0.05$, except for the survival outcome, which suffers from a limited observation interval).

Table 7 reports the discrimination performance for each clinical outcome and for each time point, i.e., the AU-ROC and the AUC-PR, computed with a 2-year timestep up to 8 years for the cardio-metabolic comorbidities and up to 4 years for survival. For each time point, the number of subjects belonging to the positive class in their actual follow-up (i.e., experiencing the outcome in reality within that time, *N positive*), the number of subjects included in the analysis (*N total*) and their ratio (*positive rate*) are also reported. Finally, in the last column, for each outcome, iAU-ROC and iAUC-PR calculated over the entire time interval are also shown. In Section S2.1 of the Supplementary material are reported the graphs of the AU-ROC and AUC-PR curves for each outcome and time point.

Both the time-dependent AU-ROC and AUC-PR measures, together with their integrated value (i.e., the iAU-ROC and iAUC-PR, respectively), range from 0 to 1, the latter indicating that all predictions are correctly ranked. The random prediction corresponds to a value of 0.5 for the AU-ROC and equal to the positive rate for the AUC-PR. We can observe that in our model the best performance is obtained for the diabetes and survival outcomes, while the other outcomes – expecially heart problems – have a more limited gain with respect to randomness.

**Table 7:** Area Under the time-dependent ROC curve (AU-ROC) and the Precision-Recall curve (AUC-PR) values computed for the cardio-metabolic comorbidities and survival on the subjects of the test set at 2, 4, 6, and 8 years since the baseline wave.

Clinical outcome		t=2	t=4	t=6	t=8	
Diabetes	AU-ROC	0.808	0.852	0.833	0.819	iAU-ROC = 0.828
	AUC-PR	0.073	0.238	0.387	0.477	iAUC-PR = 0.294
	N positive	(n=46)	(n=82)	(n=114)	(n=142)	
	N total	(N=2152)	(N=2072)	(N=1892)	(N=1447)	
	Positive rate	0.021	0.039	0.060	0.098	
Heart problems	AU-ROC	0.621	0.621	0.643	0.663	iAU-ROC = 0.637
	AUC-PR	0.039	0.102	0.187	0.298	iAUC-PR = 0.157
	N positive	(n=50)	(n=115)	(n=169)	(n=231)	
	N total	(N=1988)	(N=1935)	(N=1785)	(N=1403)	
	Positive rate	0.025	0.059	0.095	0.165	
Hypertension	AU-ROC	0.733	0.735	0.740	0.755	iAU-ROC = 0.741
	AUC-PR	0.156	0.254	0.370	0.492	iAUC-PR = 0.318
	N positive	(n=122)	(n=181)	(n=260)	(n=289)	
	N total	(N=1458)	(N=1416)	(N=1312)	(N=1034)	
	Positive rate	0.084	0.128	0.198	0.279	
Survival	AU-ROC	0.846	0.807	–	–	iAU-ROC = 0.827
	AUC-PR	0.253	0.369	–	–	iAUC-PR = 0.311
	N positive	(n=74)	(n=109)	–	–	
	N total	(N=1587)	(N=1013)	–	–	
	Positive rate	0.047	0.108	–	–	

For each clinical outcome and for each time point, the number of subjects belonging to the positive class in their actual follow-up (i.e., experiencing the outcome in reality within that time, *N positive*), the number of subjects included in the analysis (*N total*) and their ratio (*positive rate*) are reported. Finally, the last column reports the iAU-ROC and iAUC-PR calculated over the entire time interval.

### Stratification analysis

**Figure 3:**
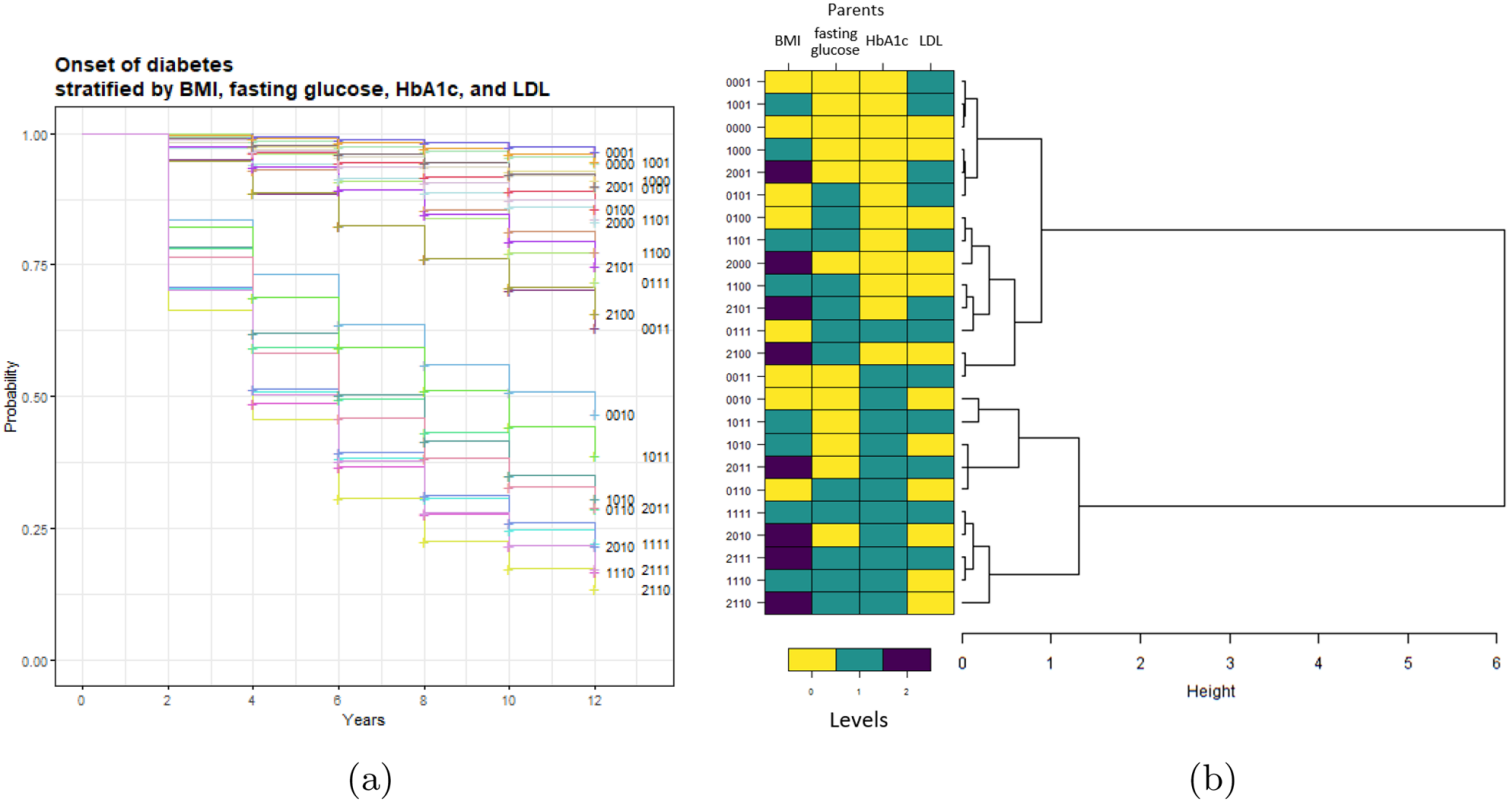
Stratification analysis of the risk of diabetes onset performed on the subjects of the training and test set. The 4-digit labels represent the quantisation level (for the 2-level variables: 0=low, 1=high; for the 3-level variables: 0=low, 1=medium, 2=high) of each parent feature of T2D at baseline, considered in the following order: BMI, fasting glucose, HbA1c, LDL. (**a**) Kaplan-Meier survival curves of the risk of diabetes onset in the different cohorts. For each cohort, the corresponding label is reported at the last value of the curve. (**b**) Dendrogram of the hierarchic agglomerative clustering performed on the Kaplan-Meier survival curves for diabetes outcome. For each cohort, the baseline values are reported on the left as a heatmap.

The main results of the stratification analysis related to the risk of developing diabetes are reported in Fig. 3. In particular, Fig. 3a shows the Kaplan-Meier curves representing the risk of diabetes in different populations which differ in the values of diabetes’ parent variables at the subjects’ baseline visit. The obtained DBN (Fig. 1) reports 4 variables as parents of the outcome of diabetes. Accordingly, each Kaplan-Meier curve is labelled with a numerical code representing the discrete values assumed by each parent variable (as reported in Table 5), considered in the following order: BMI, fasting glucose, HbA1c, LDL. For example, the curve labelled with the code *0001* is referred to those subjects who have at the baseline a BMI less than 25 $kg/m^2$, fasting glucose less than 5.6 *mmol*/*l*, HbA1c less than 6.5 % (all quantified with the value 0), and LDL greater than 1.3 mmol/l (quantified with the value 1).

Furthermore, the dendrogram resulting from the agglomerative clustering performed on these curves is shown in Fig. 3b. 10 of the 26 validity indices identify 2 as the optimal number of clusters, thus resulting in the best partition of the dendrogram. This is consistent with what we can observe from a visual inspection, where two main risk groups emerge.

Cluster 1 (C1) corresponds to a lower risk of diabetes onset (higher part of the Kaplan-Meier plot, with survival probability at 12 years above 60%), while cluster 2 (C2) corresponds to a higher risk of developing diabetes. Looking at the values assumed by each parent variable in the two clusters, we can observe that C1 is characterised by a low (i.e., category 0) HbA1c value, while C2 always presents a high (i.e., category 1) HbA1c value. By further investigating C2, we can identify 2 main sub-clusters. In detail, the one corresponding to the lower, higher-risk curves of the Kaplan-Meier plot (last 5 rows of the dendrogram) is characterised by medium/high (categories 1 and 2) values of BMI and almost always high values (category 1) of fasting glucose. On the opposite, no clear trend is observed in the LDL values with respect to the risks.

For the sake of brevity, similar analyses performed for the other two diseases, i.e., hypertension and heart problems, are reported in Section S3 of the Supplementary material.

## Discussion and conclusions

In this work, we implemented a DBN for modelling the onset and interaction of cardio-metabolic comorbidities in a population of ageing adults. After preprocessing the data according to the model requirements, we trained a network that represents the conditional dependencies among the variables over time. By analysing the emerging dependencies, we can observe how the onsets of diabetes, hypertension, and heart problems are influenced by socio-demographics characteristics, lifestyle habits, health indicators, physical measurements, and biomarkers. Through a stratification analysis, we explored the impact of variables having a major effect on the outcomes based on their values at the first subjects’ interview. Interestingly, we were able to define cohorts characterised by different risks of disease onset over time, detected both by visual inspection of the survival curves of outcome occurrence and through an automatic clustering procedure.

For this study, we employed the ELSA dataset, a large-scale longitudinal panel study that collects a large number of variables, periodically monitored in a large heterogeneous population to measure changes in their health, economic, and social circumstances. On one side, the large data cardinality and its longitudinal nature constitute a point of strength of this dataset, making it possible to observe and analyse how the population ages under a number of different aspects. On the other side, the data do not specifically focus on clinical aspects as dedicated clinical registers would do. In other words, we can observe whether a patient develops a condition, but we will not have access to many specific variables that would better describe the disease onset and care process, such as dedicated biomarkers, exams, or medications. As a further limitation, the self-reported nature of some variables that are collected through questionnaires may limit the data’s reliability. This includes the three comorbidities considered as outcomes (T2D, hypertension, and heart problems), which report patients’ response to the question “Has a doctor informed you that you have that specific condition?”. While it does not guarantee the accuracy of the diagnosis as a direct report from a doctor would, we considered this information reliable in our study. Moreover, although most subjects have been consecutively interviewed two years apart, some variables (i.e. biomarkers) have been collected every other wave only, according to the ELSA collection protocol. This different temporal resolution between the interviews and the collection of the biomarkers posed a practical problem in the design of our study. We decided to model the time by considering 2 years between the interviews, i.e., the shortest temporal distance between two consecutive collections of variables, in order to allow the detection of any changes in the health status of the subjects with the highest possible temporal resolution, thus not losing any available time points. However, this caused a high number of missing data to be present in some biomarkers. While the most desirable setting for a longitudinal analysis remains the one in which data are collected in a time-homogeneous, complete manner, some strategies can be adopted for missing data. In our case, in order to limit the missing information in the data used to learn and test the DBN, we implemented an *ad hoc* imputation procedure that takes advantage of the longitudinally available information (namely through interpolation and propagation strategies) while effectively managing the heterogeneous nature of the variables. Such an approach was considered more reasonable for the imputation of our dataset compared to other literature techniques which use the information derived from other subjects or the cross-relationships among variables to impute the missing values (e.g., weighted k-Nearest Neighbours approaches^48,49^, or the Multivariate Imputation by Chained Equations (MICE) algorithm^50^). In our case, indeed, we found a predominantly intra-subject approach to be more robust, given the nature of the missing variables (biomarkers), and chose to avoid the introduction of relational biases between variables, the detection of which was the purpose of the primary DBN-based analysis.

As a modelling technique, we have chosen DBNs, since their ability to utilise the dynamic nature of the data makes it possible to represent the entire ageing process, without focusing on a single instant in time (as more classical regression or classification methods would do), but rather by actually keeping track of the time that passes from a baseline. For doing that, we introduced the TSB variable that marks the time in the data and allows the DBN to compute the conditional dependencies on different time slices, thus modelling in a possibly different manner how the relationships among variables mutate as the population ages. As for the implementation of the DBN, we selected the discrete-time, discrete-space one available in the *bnstruct* package, which allowed us to handle mixed data by discretising the continuous variables. Although effective, this strategy has the limitation of reducing the resolution of the information originally contained in the continuous variables. Indeed, for this type of features, the network learns on the quantised/discretised levels of the variables, each representing the combined contribution of all the continuous values falling within it. For what concerns the simulation (see Section 3.2), the network also provides as an outcome the discretised value of each variable, simulated at subsequent time points starting from the value of its parents at the previous time point. In case the predicted values are of interest on a continuous scale, our procedure allows to de-quantise them by extracting a value from the distribution of the continuous values of the corresponding discrete level. This strategy, although possibly leading to an approximation of the predicted values due to the indirect simulation of the continuous value, effectively returns a value within the predicted discrete range and proportionally sampled from the values observed in the training dataset. In the case of the outcomes considered for this study, however, since their levels are binary by definition (being the occurrence or absence of the onset of a comorbidity or the survival), no possible artefacts due to de-quantification were introduced into their prediction.

As reported in Table 7 and Fig. 2, the obtained model presents a good overall discrimination and calibration ability on the test set. Specifically, higher discrimination performance is obtained when predicting the onset of type 2 diabetes (iAU-ROC = 0.828, iAUC-PR = 0.294) and the survival (iAU-ROC = 0.827, iAUC-PR = 0.311), while it lowers for hypertension and heart problems, with an iAU-ROC equal to 0.741 and 0.637 and an iAUC-PR equal to 0.318 and 0.157, respectively. This reflects the trends observed in the training set (see Section S2.2 of the Supplementary Material, Table S1). Moreover, a good calibration ability is shown in the comorbidities risk prediction, as confirmed by the log-rank test which did not find any significant difference between real and simulated Kaplan-Meier curves (p-value>0.05). The model was found to be less calibrated only for death, a fact that can be due to the limited available observation interval. These findings align with the observations made in the training set (see Section S2.2 of the Supplementary Material, Figure S6).

DBNs represent a highly interpretable tool for modelling the evolution of a number of conditions at the same time, enabling us to easily understand how the variables interact with each other. The combined analysis of the relationships among the variables reported in the DAG together with their conditional probabilities provides a complete overview of how the patients’ evolution was changing over time. Specifically, the DAG highlights the set of variables which have a combined direct effect on a child variable, as well as the features which influence it indirectly; the study of the CPTs, which have here been exploited in the simulation procedure and stratification analysis, permits the investigation of the direction of the relationships (for example, if a higher value of a parent variable, holding the value of the other parents fixed, causes the value of the child variable to increase or decrease).

By analysing the network learned in this work with respect to the cardio-metabolic comorbidities, we can observe how the development of a morbidity is often related to two different categories of variables: the first group includes biomarkers clinically associated with the definition of the disease (e.g., high levels of fasting glucose and HbA1c at time *t-1* are related to an enhanced probability of developing diabetes at time *t*); the second group includes lifestyle habits or health indicators that are known as risk factors (such as higher BMI value on the development of diabetes). In contrast, no direct cross-relationships between the comorbidities emerge, being rather mediated by other variables.

The exploration of comorbidities is a topic of significant interest, with numerous ongoing studies and a broad literature published on the subject. To the best of our knowledge, however, literature studies do not focus on the longitudinal development and possible interaction between diseases, tending instead to analyse risk factors and onset of individual diseases, making limited use of longitudinal variables. ELSA data have already been analysed in previous literature works with the aim of highlighting strong/known risk factors for the development of cardio-metabolic comorbidities; however, compared to our work, all these literature works have focused on a single disease at a time, rather than considering several diseases in the same model. Analysing the relationships highlighted in previous works on the ELSA dataset and comparing them to our analyses, we find again the association of BMI (or waist) and cholesterol indicators with the development of diabetes^18,51^, of BMI with hypertension^16^, and of age with heart problems^52^. Less investigated in the literature, however, is the impact of health indicators such as ADL, with which a strong association was found in this work with both hypertension and heart problems. Therefore, although some of the observed network dependencies are already known in the literature, the ability of the developed DBN to show how chronic diseases and risk factors interact, both as direct and indirect links, represents a novelty compared to what can be achieved by more traditional statistical methods.

In addition, the stratification analysis we performed based on the hierarchical agglomerative clustering applied to the Kaplan-Meier simulated curves allowed us to better investigate the effect of the parent variables on the development of cardio-metabolic comorbidities. For each outcome, we identified in an automatic way clusters characterised by similar risk profiles: for diabetes (see Fig. 3), the population was clearly divided into two groups, while for the other comorbidities the distinction was not as clear from a visual inspection only (see Section S7 in the Supplementary Material). Similar final identified clusters have been obtained by using the average clusters agglomeration method compared to the Ward’s criterion (on which are based the results presented in this work). By analysing the values of the outcome’s parents at baseline, we were also able to detect variables that suggest a clear discriminating effect among the clusters on their own, such as HbA1c for diabetes or age at baseline for heart problems; in contrast, some other variables do not show an equally clear discriminating ability. However, since they were selected as parents by the DBN, their contribution has to be intended as a joint effect together with the other parents.

An interesting future work could be the validation of the DBN on a different population. Interestingly, the model presented in this work could not only be compared to a new DBN developed on other data, but also directly updated by using the new dataset for recomputing the CPTs while maintaining the network structure. Moreover, the tool could also be expanded by including other chronic diseases, such as mental conditions.

To conclude, the developed DBN represents a valuable risk tool for the study of cardio-metabolic comorbidities development and interaction based on socio-demographic, clinical, and biomarkers variables. Compared to other approaches, a DBN-based study yields results that are highly interpretable during the analysis phase and easily communicated, thanks to the graphical representation of the relationships found. The simulation and clustering studies implemented in this work, moreover, make it possible to validate and exploit the model to better study the risk of occurrence of the diseases considered, indicating the most impactful worsening or prevention factors. Studies on this topic are of pivotal importance for gaining knowledge aimed at designing prevention strategies and targeted interventions for improving the population’s health status. Models and stratification analyses such as the ones proposed in this paper could serve as tools to identify the factors contributing to the development of cardio-metabolic comorbidities, identifying their direction on the risk of onset and quantifying their impact on the subject’s health in a wide sense. From a preventive point of view, the proposed tool can be used to simulate in silico the effect of different disease management guidelines (such as diet and sport in pre-diabetic subjects) or active ageing strategies, testing their effect in the short, medium, and long term on a heterogeneous or selected population. In this way, the developed model could be used to support public health and policy-makers in health policy planning and decision-making.

### Supplementary Information

Supplementary Information.

## Data Availability

The ELSA dataset is available here: https://www.elsa-project.ac.uk/. The model presented in this work has been implemented as an R code available from the corresponding author on request.

## Abbreviations

ADL: Activities of daily living
AU-ROC: Area under the receiving operating characteristic curve
BIC: Bayesian information criterion
BMI: Body mass index
BP: Blood pressure
CAPI: Computer-assisted personal interviewing
CPTs: Conditional probability tables
CRP: C-reactive protein
CV: Cross-validation
C1: Cluster 1
C2: Cluster 2
C3: Cluster 3
DAG: Directed acyclic graph
DBN: Dynamic Bayesian network
ELSA: English longitudinal study of ageing
HbA1c: Glycated hemoglobin
HDL: High-density lipoprotein
IADL: Instrumental activity of daily living
iAU-ROC: integrated AUC-ROC
LDL: Low-density lipoprotein
LOCF: Last observation carried forward
MMHC: Maximum minimum hill climbing
NOCB: Next observation carried backward
NRES: National research and ethics service
PAPI: Pen-and-paper personal interview
TSB: Time since baseline
T2D: Type 2 diabetes
WPDAG: Weighted partially directed acyclic graph
