# Supplementary material for: A dynamic probabilistic model of the onset and interaction of cardio-metabolic comorbidities on an ageing adult population
Source: Sci Rep. 2024 May 20;14:11514. doi: 10.1038/s41598-024-61135-x (PMC11106085; doi:10.1038/s41598-024-61135-x)
Supplement: Supplementary file 1 — Supplementary Information. [file 41598_2024_61135_MOESM1_ESM.pdf]

A Dynamic Probabilistic Model of the Onset and Interaction of Cardio-metabolic Comorbidities on an Ageing Adult Population
Supplementary Material

Chiara Roversi[1,*], Erica Tavazzi[1,*], Martina Vettoretti[1], Barbara Di Camillo[1,2,§]

[1] University of Padua, Department of Information Engineering
[2] University of Padua, Department of Comparative Biomedicine and Food Science

[*] These authors contributed equally.
[§] Correspondence to: barbara.dicamillo@unipd.it

S1 Network learning

S1.1 Starting network obtained through a 50-fold CV process on the training set

As mentioned in Section 2.2.1 of the Manuscript, we performed a 50-fold cross-validation procedure on the training set to obtain a DAG to be provided as the starting network in the learning phase. Figure S1 reports the resulting DAG, constituted by the edges resulting in at least 80% of the CV-trained DAGs.

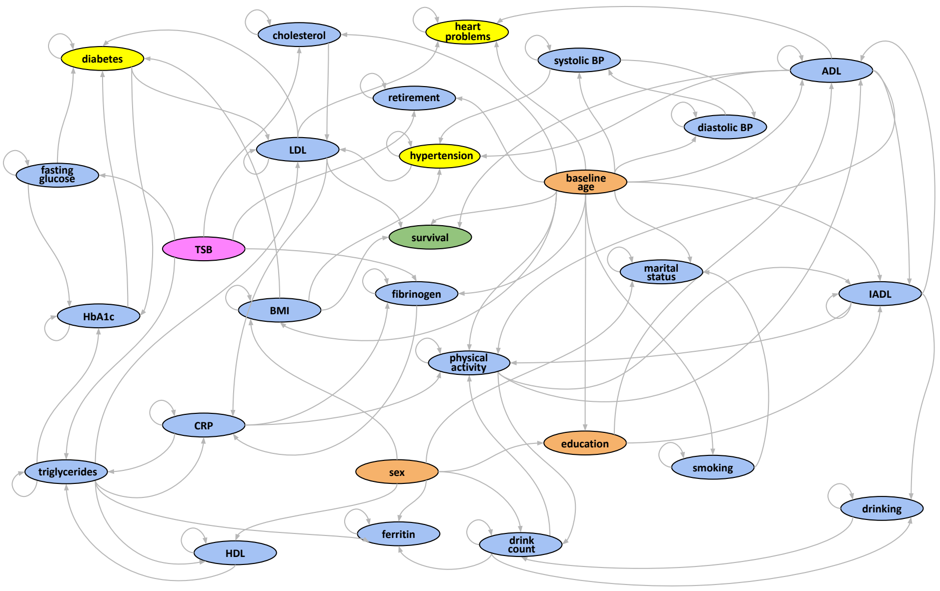

Figure S1: WPDAG obtained through the CV procedure in the training set and used as the initial network in the final learning process of the DBN. Static variables are reported in orange, dynamic variables in blue, the time variable in magenta, the survival in green, while the outcomes, i.e. T2D, hypertension, and heart problems onset, in yellow. The loops on the dynamic variables indicate the influence of the variable at time (t-1) on itself at subsequent time (t). Only the edges that occur in at least 80% of the CV-trained DAGs are shown.

With respect to this network provided as the starting point, we can observe that in the final DBN the following edges appear:

- fibrinogen → survival
- IADL → survival
- diabetes → cholesterol
- drinking → IADL
- smoking → drink count
- sex → smoking
- CRP → HbA1c
- HbA1c → fasting glucose

S2 Network performance

S2.1 Network performance on the training set

Figures S2, S3, S4, and S5 report the time-dependent Receiver Operating Characteristic (ROC) and the Precision-Recall (PR) curves on the test set for the diabetes, heart problems, hypertension, and survival outcome, respectively. The curves are computed with a 2-year timestep up to 8 years for the cardio-metabolic comorbidities and up to 4 years for survival. For both ROC and PR, the value of the area under the curve is also reported. For the PR curves, the dashed horizontal line represents the performance of the random model, equal to the positive rate.

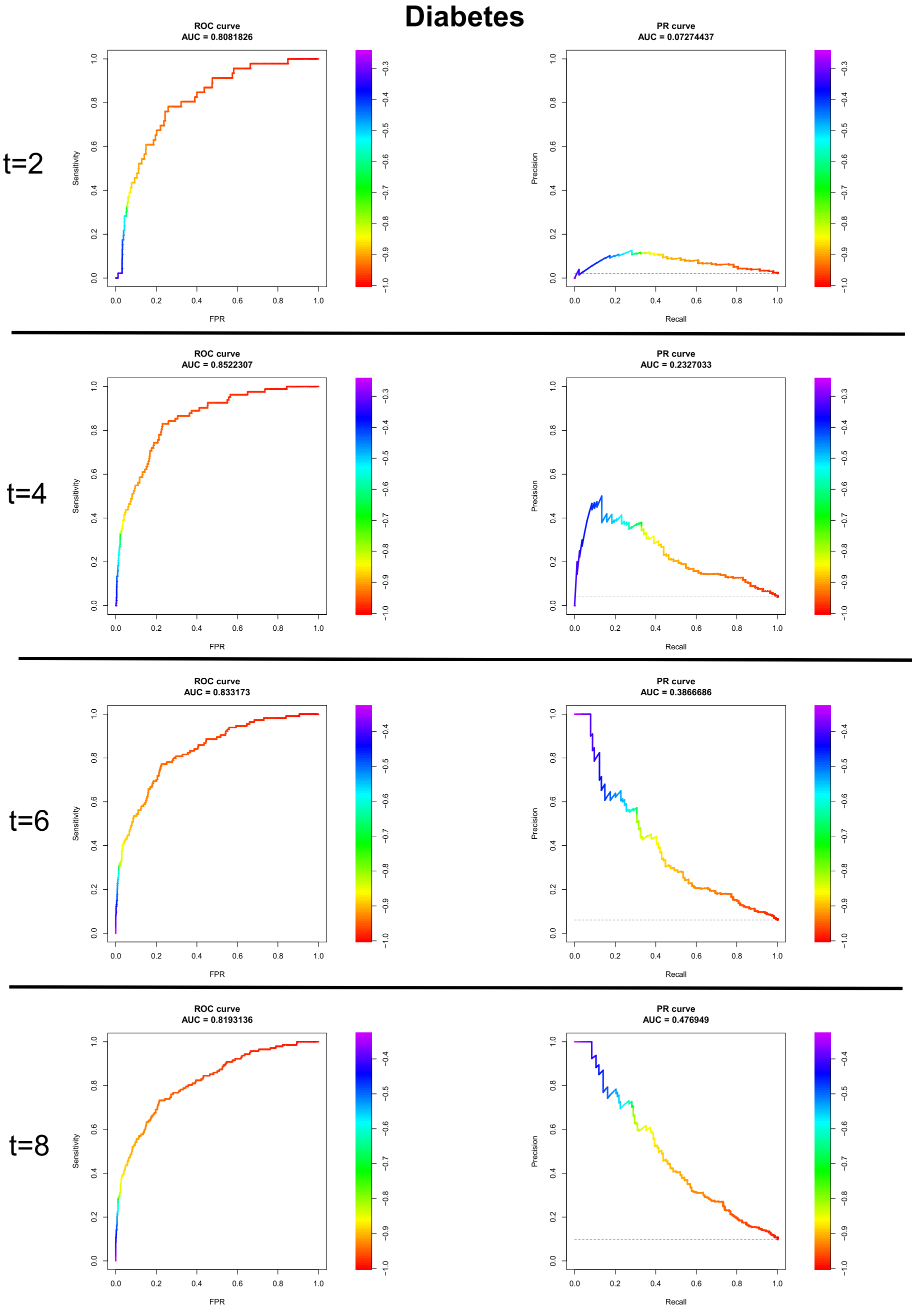

Figure S2: Receiver Operating Characteristic (ROC, on the left column) and Precision-Recall (PR, on the right column) curves calculated on the test set for the diabetes outcome, calculated with a time step of t=2 years up to 8 years (one row for each). For both ROC and PR, the value of the area under the curve is also reported. For the PR curves, the dashed horizontal line represents the performance of the random model, equal to the positive rate.

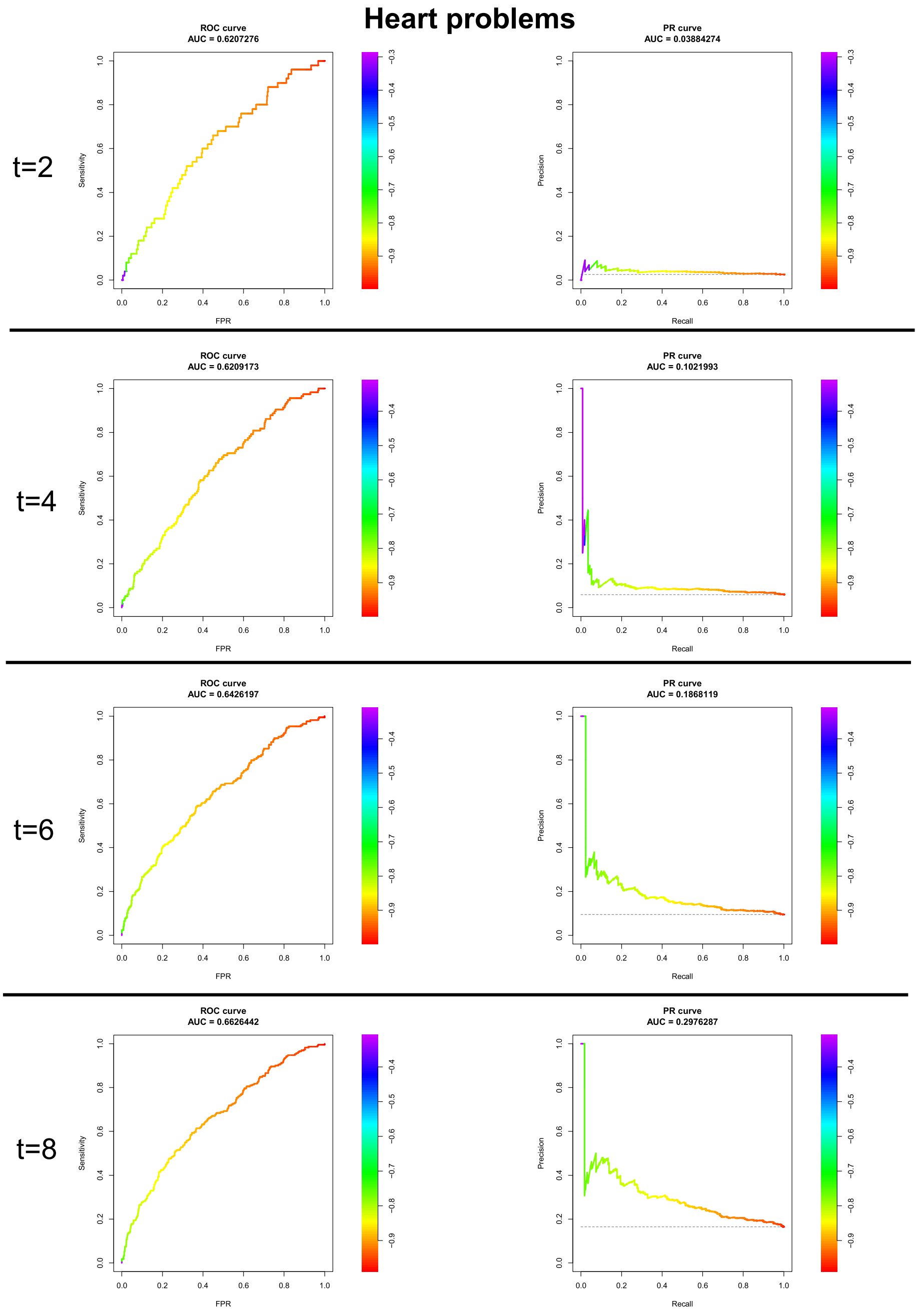

Figure S3: Receiver Operating Characteristic (ROC, on the left column) and Precision-Recall (PR, on the right column) curves calculated on the test set for the heart problem outcome, calculated with a time step of t=2 years up to 8 years (one row for each). For both ROC and PR, the value of the area under the curve is also reported. For the PR curves, the dashed horizontal line represents the performance of the random model, equal to the positive rate.

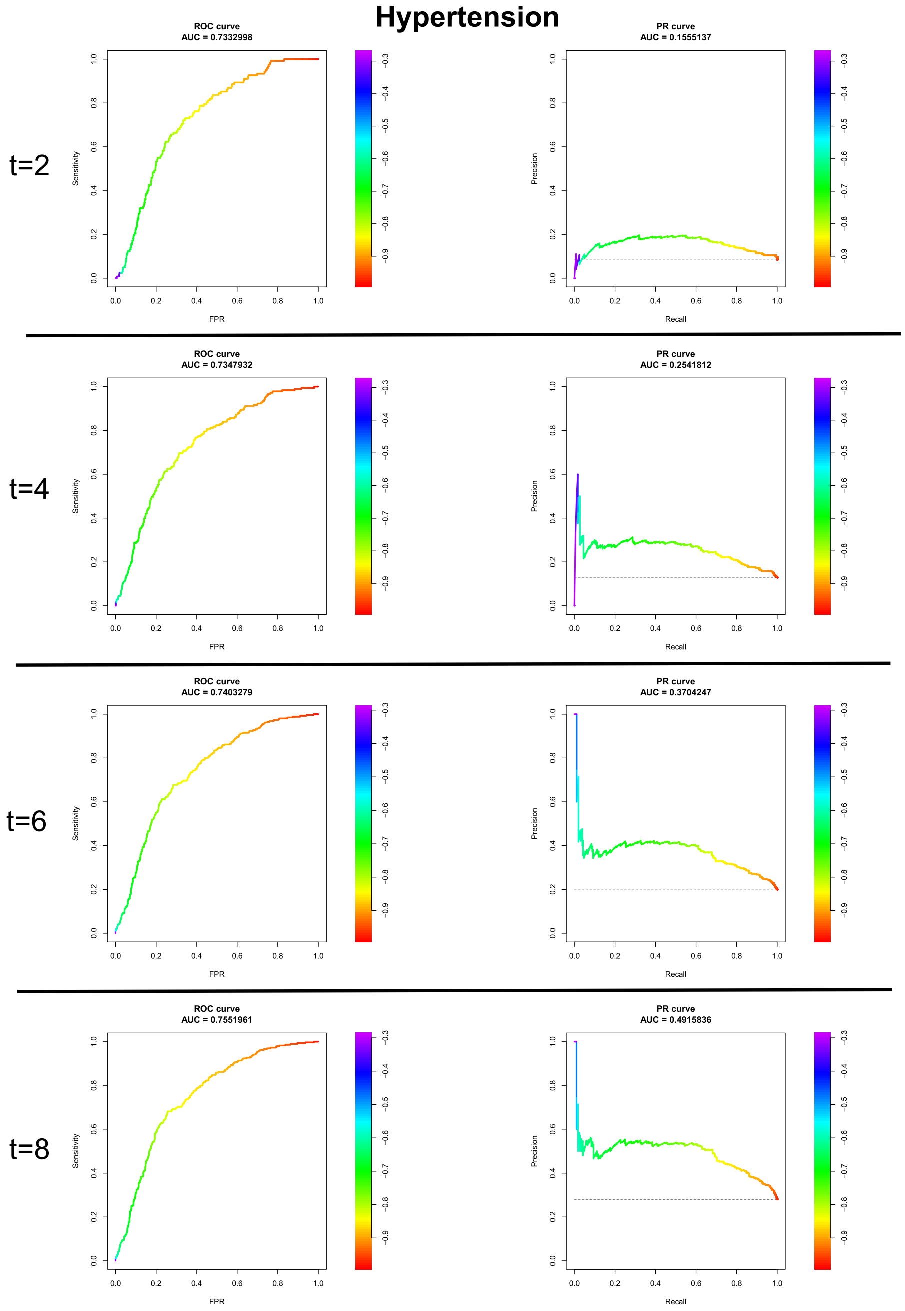

Figure S4: Receiver Operating Characteristic (ROC, on the left column) and Precision-Recall (PR, on the right column) curves calculated on the test set for the hypertension outcome, calculated with a time step of t=2 years up to 8 years (one row for each). For both ROC and PR, the value of the area under the curve is also reported. For the PR curves, the dashed horizontal line represents the performance of the random model, equal to the positive rate.

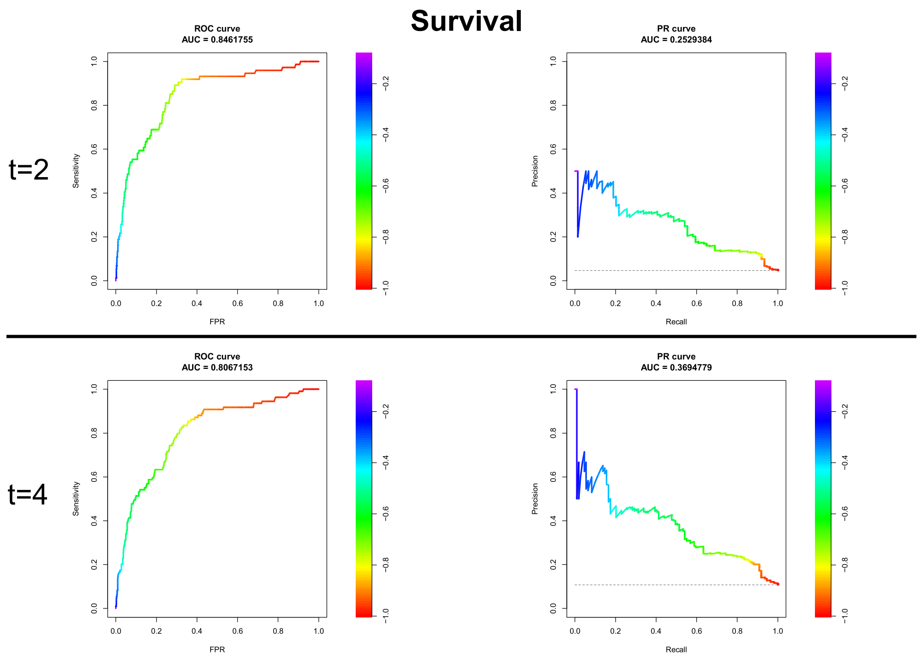

Figure S5: Receiver Operating Characteristic (ROC, on the left column) and Precision-Recall (PR, on the right column) curves calculated on the test set for the survival outcome, calculated with a time step of t=2 years up to 4 years (one row for each). For both ROC and PR, the value of the area under the curve is also reported. For the PR curves, the dashed horizontal line represents the performance of the random model, equal to the positive rate.

S2.2 Network performance on the training set

As reported for the test set in section 3.2 of the Manuscript, here we report the DBN performance on the training set.

Figure S6 reports the time of occurrence for the real and simulated outcomes computed in terms of the Kaplan-Meier estimator in the population of the training set, reduced to the only subjects with a complete first visit (n=6935). Since in the real population the outcomes also occur at odd years (being the distance between two consecutive real waves only approximately equal to 2 years), the visual comparison among the real and simulated Kaplan-Meier curves has to be limited to the common, even time points. Together with the visual inspection, the log-rank test quantitatively confirms that the model is in general well calibrated (all p-values > 0.05, except for the survival outcome, which suffers from a limited observation interval).

Table S1 reports the discrimination performance for each clinical outcome and for each time point on the training set, i.e., the AU-ROC and the AUC-PR, computed with a 2-year timestep up to 8 years for the cardiometabolic comorbidities and up to 4 years for survival. For each time point, the number of subjects belonging to the positive class in their actual follow-up (i.e., experiencing the outcome in reality within that time, *N positive*), the number of subjects included in the analysis (*N total*) and their ratio (*positive rate*) are also reported. Finally, in the last column, for each outcome, iAU-ROC and iAUC-PR calculated over the entire time interval are also shown.

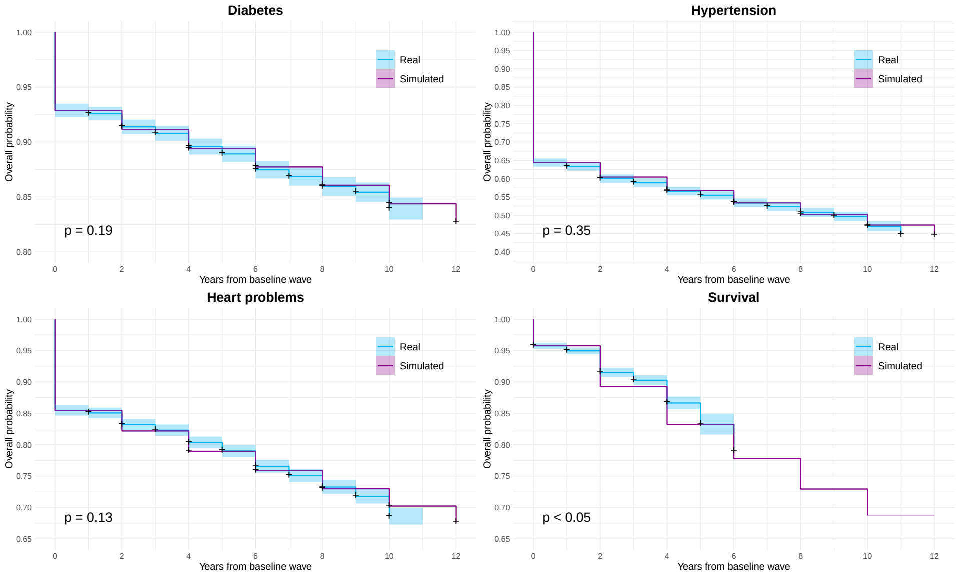

Figure S6: Kaplan-Meier curves of cardio-metabolic comorbidities onset and survival for the training data. Shaded areas denote confidence intervals ($\alpha = 0.05$), + indicates censored subjects. For each outcome, the log-rank test's p-value is reported, with significance threshold set equal to 0.05.

Table S1: Area Under the time-dependent ROC curve (AU-ROC) and the Precision-Recall curve (AUC-PR) values computed for the cardio-metabolic comorbidities and survival on the subjects of the training set at 2, 4, 6, and 8 years since the baseline wave. For each clinical outcome and for each time point, the number of subjects belonging to the positive class in their actual follow-up (i.e., experiencing the outcome in reality within that time, *N positive*), the number of subjects included in the analysis (*N total*) and their ratio (*positive rate*) are reported. Finally, the last column reports the iAU-ROC and iAUC-PR calculated over the entire time interval.

**Clinical outcome**		**t=2**	**t=4**	**t=6**	**t=8**	
**Diabetes**	**AU-ROC**	0.816	0.811	0.791	0.792	**iAU-ROC** = 0.802
	**AUC-PR**	0.079	0.247	0.382	0.491	**iAUC-PR** = 0.299
	**N positive**	(n=103)	(n=222)	(n=352)	(n=421)	
	**N total**	(N=6382)	(N=6136)	(N=5616)	(N=4129)	
	**positive rate**	0.016	0.036	0.063	0.102	
**Heart problems**	**AU-ROC**	0.632	0.631	0.631	0.643	**iAU-ROC** = 0.634
	**AUC-PR**	0.055	0.127	0.210	0.308	**iAUC-PR** = 0.175
	**N positive**	(n=157)	(n=348)	(n=587)	(n=740)	
	**N total**	(N=5886)	(N=5718)	(N=5302)	(N=3984)	
	**positive rate**	0.027	0.061	0.111	0.186	
**Hypertension**	**AU-ROC**	0.699	0.719	0.725	0.723	**iAU-ROC** = 0.717
	**AUC-PR**	0.128	0.272	0.381	0.496	**iAUC-PR** = 0.319
	**N positive**	(n=303)	(n=530)	(n=731)	(n=850)	
	**N total**	(N=4435)	(N=4298)	(N=4010)	(N=3070)	
	**positive rate**	0.068	0.123	0.182	0.277	
**Survival**	**AU-ROC**	0.802	0.805	–	–	**iAU-ROC** = 0.803
	**AUC-PR**	0.167	0.356	–	–	**iAUC-PR** = 0.262
	**N positive**	(n=207)	(n=365)	–	–	
	**N total**	(N=4617)	(N=2970)	–	–	
	**positive rate**	0.045	0.123	–	–	

S3 Stratification analysis

S3.1 Stratification analysis: heart problems

Figure S7a shows the Kaplan-Meier curves representing the risk of heart problems in different populations that differ in the values of the heart problems' parent variables at the subjects' baseline visit. The obtained DBN (see Fig. 1 of the Manuscript) reports 3 variables as the parents of the heart problems outcome. Accordingly, each Kaplan-Meier curve is labelled with a numerical code that represents the discrete values assumed by each parent variable (as reported in Table 5 of the Manuscript), considered in the following order: baseline age, ADL, and LDL.

The dendrogram resulting from the agglomerative clustering performed on these curves is shown in Fig. S7b. 12 of the 26 validity indices identify 2 as the optimal number of clusters, thus resulting in the best partition of the dendrogram. Cluster 1 (C1) corresponds to a lower risk of developing heart problems (higher part of the Kaplan-Meier plot, with survival probability at 12 years above 83%), while cluster 2 (C2) corresponds to an enhanced risk. As the main discriminating variable, we find age at baseline, whose higher

values characterise C2.

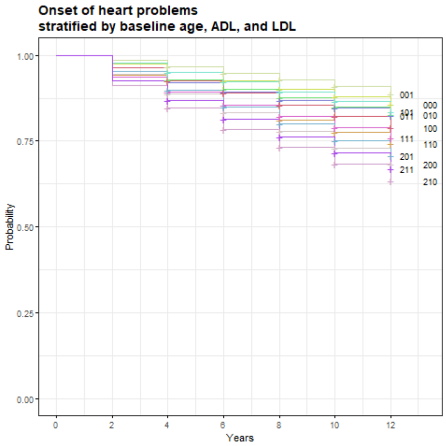

(a) Kaplan-Meier survival curves of the risk of heart problems onset in the different cohorts. For each cohort, the corresponding label is reported at the last value of the curve.

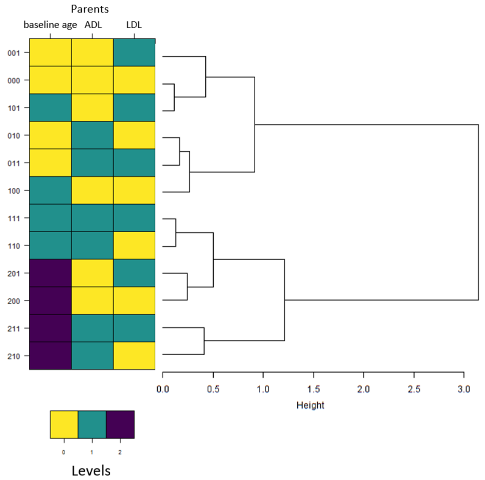

(b) Dendrogram of the hierarchic agglomerative clustering performed on the Kaplan-Meier survival curves for heart problems outcome. For each cohort, the baseline values are reported on the left as a heatmap.

Figure S7: Stratification analysis of the risk of heart problems onset performed on the subjects of the training and test set. The 3-digit labels represent the quantisation level (for the 2-level variables: 0=low, 1=high; for the 3-level variables: 0=low, 1=medium, 2=high) of each parent feature of heart problems at baseline, considered in the following order: age at baseline, ADL, LDL.

S3.2 Stratification analysis: hypertension

Figure S8a shows the Kaplan-Meier curves representing the risk of hypertension in different populations that differ in the values of the hypertension's parent variables at the subjects' baseline visit. The obtained DBN (see Fig. 1 of the Manuscript) reports 3 variables as parents of the hypertension outcome. Accordingly, each Kaplan-Meier curve is labelled with a numerical code representing the discrete values assumed by each parent variable (as reported in Table 5 of the Manuscript), considered in the following order: ADL, BMI, and systolic BP.

The dendrogram resulting from the agglomerative clustering performed on these curves is shown in Fig. S7b. 8 of the 26 validity indices identify 2 as the optimal number of clusters, thus resulting in the best partition of the dendrogram. Cluster 1 (C1) corresponds to a lower risk of developing hypertension (higher part of the Kaplan-Meier plot, with survival probability at 12 years above 55%), while cluster 2 (C2) corresponds to an enhanced risk. As the main discriminating variable, we find the value of the systolic pressure at the previous wave, whose higher values, as expected, correspond to a higher risk of developing hypertension.

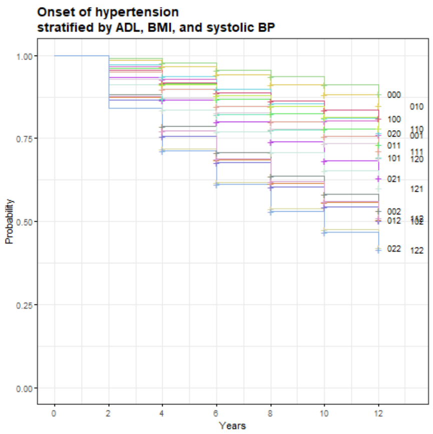

(a) Kaplan-Meier survival curves of the risk of hypertension onset in the different cohorts. For each cohort, the corresponding label is reported at the last value of the curve.

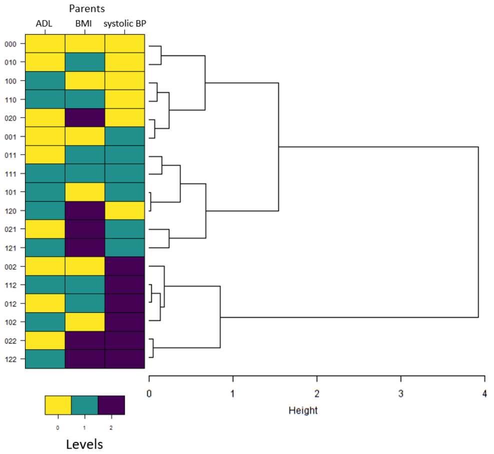

(b) Dendrogram of the hierarchic agglomerative clustering performed on the Kaplan-Meier survival curves for hypertension outcome. For each cohort, the baseline values are reported on the left as a heatmap.

Figure S8: Stratification analysis of the risk of hypertension onset performed on the subjects of the training and test set. The 3-digit labels represent the quantisation level (for the 2-level variables: 0=low, 1=high; for the 3-level variables: 0=low, 1=medium, 2=high) of each parent feature of hypertension at baseline, considered in the following order: ADL, BMI, systolic BP.
